# Effects of Size and Aggregation/Agglomeration of Nanoparticles on the Interfacial/Interphase Properties and Tensile Strength of Polymer Nanocomposites

**DOI:** 10.1186/s11671-018-2624-0

**Published:** 2018-07-17

**Authors:** Muhammad Aqeel Ashraf, Wanxi Peng, Yasser Zare, Kyong Yop Rhee

**Affiliations:** 1 School of Forestry, Henan Agricultural University, Zhengzhou, 450002 China; 2 Faculty of Science, Department of Geology, University of Malaya, 50603 Kuala Lumpur, Malaysia; 3 Young Researchers and Elites Club, Science and Research Branch, Islamic Azad University, Tehran, Iran; 4 Department of Mechanical Engineering, College of Engineering, Kyung Hee University, Yongin, 446-701 Republic of Korea

**Keywords:** Polymer nanocomposites, Particle size, Aggregation/agglomeration, Interfacial/interphase properties

## Abstract

In this study, several simple equations are suggested to investigate the effects of size and density on the number, surface area, stiffening efficiency, and specific surface area of nanoparticles in polymer nanocomposites. In addition, the roles of nanoparticle size and interphase thickness in the interfacial/interphase properties and tensile strength of nanocomposites are explained by various equations. The aggregates/agglomerates of nanoparticles are also assumed as large particles in nanocomposites, and their influences on the nanoparticle characteristics, interface/interphase properties, and tensile strength are discussed. The small size advantageously affects the number, surface area, stiffening efficiency, and specific surface area of nanoparticles. Only 2 g of isolated and well-dispersed nanoparticles with radius of 10 nm (*R* = 10 nm) and density of 2 g/cm^3^ produce the significant interfacial area of 250 m^2^ with polymer matrix. Moreover, only a thick interphase cannot produce high interfacial/interphase parameters and significant mechanical properties in nanocomposites because the filler size and aggregates/agglomerates also control these terms. It is found that a thick interphase (*t* = 25 nm) surrounding the big nanoparticles (*R* = 50 nm) only improves the *B* interphase parameter to about 4, while *B* = 13 is obtained by the smallest nanoparticles and the thickest interphase.

## Background

The nanocomposites exhibit substantial properties by only small content of nanofiller [1–5]. The important properties of polymer nanocomposites cause a wide range of applications in various technologies such as advanced materials and goods, medicines, energy devices, and sensors [6]. The studies on different types of polymer nanocomposites aim to achieve high-performance products by an easy fabrication process and low cost.

The considerable properties of polymer nanocomposites are attributed to good interfacial properties between polymer matrix and nanoparticles such as interfacial area and interaction/adhesion at interface [7–13]. The high levels of interfacial properties lead to formation of another phase as interphase around the nanoparticles which is different from both polymer matrix and nanoparticles shows the advantage of nanocomposites compared to conventional micro-composites [14–18]. Many theoretical investigations on interfacial/interphase properties have given a large amount of information to attain the desirable properties. However, the high surface area of nanoparticles and the strong attractive interaction between particles result in the aggregation/agglomeration [19, 20]. The strong and dense collectives of nanoparticles denote the aggregation, but the loosely joint particles show the agglomeration which may be broken by mechanical stress [21].

The aggregation/agglomeration of nanoparticles reduces the potential enhancement of mechanical properties in nanocomposites, due to the restriction of interfacial area [22, 23]. Therefore, the main challenge in production of nanocomposites includes the achievement of small nanoparticles and good dispersion of nanoparticles. It is vital to overcome the attractive forces between nanoparticles producing the aggregation/agglomeration, instead of disturbing the structure of nanoparticles. Surprisingly, Dorigato et al. [24] suggested a model which shows the primary filler aggregation reinforces the polymer nanocomposites, while the agglomerated nanoparticles commonly induce negative effects on the mechanical performances of polymer nanocomposites [21, 25]. Accordingly, the study on aggregation/agglomeration of nanoparticles is required to reveal its real effects on the properties of nanocomposites. Although the nanoparticle size is assumed as an attractive benefit in polymer nanocomposites, the effects of isolation or aggregation/agglomeration on the main properties of nanoparticles such as number, surface area, and specific surface area have not been studied in the literature. Moreover, the aggregation/agglomeration of nanoparticles has been assumed as a general term which qualitatively changes the behavior of nanocomposites. Also, the possible roles of nanoparticle and interphase dimensions on the interfacial/interphase properties have not been described in previous studies.

## Methods

In this paper, the effects of filler size and density on the number, surface area, stiffening efficiency, and specific surface area of nanoparticles in polymer nanocomposites are explained by proper equations. Also, the aggregation/agglomeration of nanoparticles is assumed as large particles and their influences on various terms are revealed. Similarly, the possible roles of nanoparticle and interphase sizes in the interfacial/interphase parameters and tensile strength of nanocomposites are discussed. The main focus of this article is on the spherical nanoparticles, but other nanoparticle geometries can be studied by development of the suggested equations.

The number of spherically isolated nanoparticles in a nanocomposite can be calculated by the weight of nanoparticles (*W*_*f*_) as:

1 $$ N=\frac{W_f}{d_f\frac{4}{3}\pi {R}^3}. $$

where *d*_*f*_ and *R* are the density and radius of nanoparticles, respectively. In this condition, the total surface area of dispersed nanoparticles is given by:

2 $$ A=N\left(4\pi {R}^2\right). $$

*A* can be considered as the interfacial area between polymer matrix and nanoparticles. Replacing of *N* from Eq. 1 into Eq. 2 leads to:

3 $$ A=\frac{3{W}_f}{d_fR}. $$

which correlates the *A* with *W*_*f*_, *d*_*f*_, and *R*.

Each nanoparticle introduces a stiffening effect in polymer matrix by mechanical involvement of polymer chains. The level of stress sharing between polymer matrix and nanoparticles depends on the interfacial area and the stiffness of nanoparticles. As a result, a novel parameter as the stiffening efficiency of nanoparticles can be defined as:

4 $$ SE={AE}_f=\frac{3{W}_f}{d_fR}{E}_f. $$

where *E*_*f*_ is the Young’s modulus of nanoparticles. The stiffening efficiency as a function of the properties of nanoparticles expresses the capability of nanoparticles for the stiffening of nanocomposites. Additionally, the specific surface area of particles is expressed as:

5 $$ {A}_c=\frac{A}{m}=\frac{A}{d_fv}=\frac{4\pi {R}^2}{d_f\frac{4}{3}\pi {R}^3}=\frac{3}{d_fR}. $$

where *m* and *v* are total mass and volume of nanoparticles, respectively. This parameter expresses the surface area of 1 g particles and so, does not depend on the concentration of nanoparticles in nanocomposite.

Now, the tensile strength and interfacial/interphase properties are given by simple equations. Pukanszky [26] suggested a model for tensile strength of composites as a function of filler content and interfacial/interphase properties as:

6 $$ \sigma ={\sigma}_m\frac{1-{\varphi}_f}{1+2.5{\varphi}_f}\exp \left(B{\varphi}_f\right). $$

where *σ*_*m*_ shows the tensile strength of polymer matrix and *φ*_*f*_ is the volume fraction of nanofiller. This model was originally suggested for composites, but this model has shown good agreements with the experimental results of different polymer nanocomposites. A good agreement is obtained between the experimental data of tensile strength and the predictions of Pukanszky equation in many samples such as PP/SiO_2_ [27], PEEK/SiO_2_ [28], PVC/CaCO_3_ [29], PP/CaCO_3_ [30], and PVC/SiO_2_ [31] calculating the *B* parameter as 4.12, 3.15, 3.07, 2.5, and 2.1, respectively. These examples validate the application of Pukanszky model for the tensile strength of polymer nanocomposites.

*B* is an interfacial parameter which shows the level of interfacial adhesion by:

7 $$ B=\left(1+{A}_c{d}_ft\right)\ln \left(\frac{\sigma_i}{\sigma_m}\right). $$

where *t* and *σ*_*i*_ are the thickness and strength of interphase, respectively.

Replacing of *A*_*c*_ from Eq. 5 into the latter equation presents:

8 $$ B=\left(1+3\frac{t}{R}\right)\ln \left(\frac{\sigma_i}{\sigma_m}\right). $$

Applying the above equation into Pukanszky model offers the relative strength (*σ*/*σ*_*m*_) as:

9 $$ {\sigma}_R=\frac{1-{\varphi}_f}{1+2.5{\varphi}_f}\exp \left[\left(1+3\frac{t}{R}\right)\ln \left(\frac{\sigma_i}{\sigma_m}\right){\varphi}_f\right]. $$

which explicitly links the tensile strength to filler and interphase properties. Also, we should indicate the size effects, which undoubtedly exist when modeling fracture [32–34].

The volume fraction of interphase (*φ*_*i*_) for nanocomposites containing spherical nanoparticles can be considered [35] by:

10 $$ {\varphi}_i=\left[{\left(\frac{R+t}{R}\right)}^3-1\right]{\varphi}_f. $$

in which *t* = 0 results in *φ*_*i*_ = 0 indicating the absence of interphase in nanocomposite. The analytical models in this study may be applicable where other models such as cohesive zone describe the interphase regions. Some previous studies have considered the interphase by some models such as 2D finite element [36, 37].

In our previous work [38], *a* interphase parameter for polymer nanocomposites reinforced with spherical nanoparticles was defined as:

11 $$ a=10\left(\frac{t}{R}\right)\left(\frac{10{E}_i}{E_f}-1\right). $$

where *E*_*i*_ is the modulus of interphase. This equation correlates the *a* to various effective parameters of nanofiller and interphase. *a* was calculated for some nanocomposites ranging from 0.8 to 19 [38]. It was reported that a higher level of *a* introduces a better modulus in nanocomposite.

## Results and discussion

At the first part of this section, the effects of size and density on different properties of nanoparticles are plotted by contour plots and the results are discussed to clarify the influence of aggregation/agglomeration. At the next step, the roles of nanoparticle radius (including the aggregation/agglomeration) and interphase thickness in the interfacial/interphase properties and nanocomposite performances are studied.

**Fig. 1:**
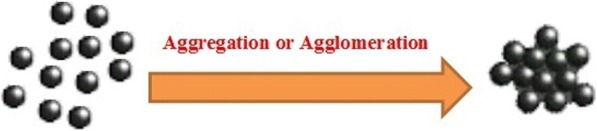
Schematic illustration of aggregation/agglomeration of nanoparticles in polymer nanocomposites. When several nanoparticles with radius *R* are aggregated/agglomerated, a large particle is formed

Figure 1 illustrates the aggregation/agglomeration of nanoparticles in a nanocomposite. When the isolated and dispersed nanoparticles are accumulated, it can be assumed that a large nanoparticle is formed. According to Fig. 1, if isolated nanoparticles with *R* radius aggregate/agglomerate, a big particle is produced with high radius. As a result, the aggregation/agglomeration of nanoparticles can be physically assumed by growth of particle size in nanocomposites. This occurrence affects the characteristics of nanoparticles and interphase which finally change the behavior of nanocomposites.

**Fig. 2:**
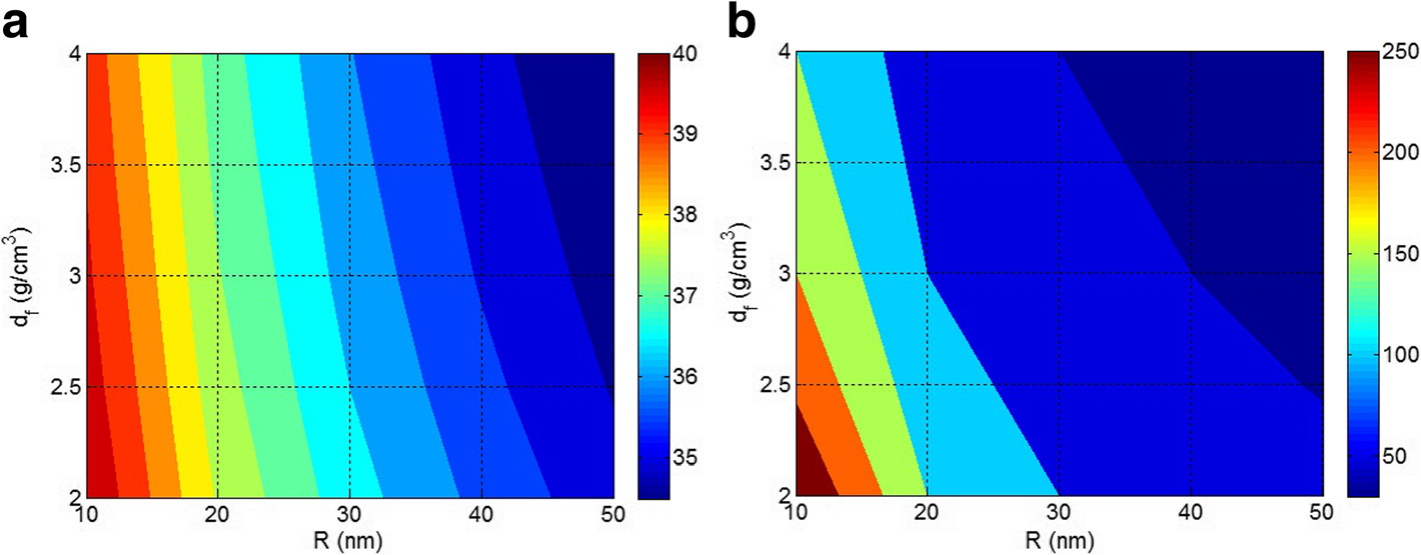
Contour plots to show the roles of *R* and *d*_*f*_ parameters in **a** ln (*N*) and **b** *A* (m^2^) at *W*_*f*_ = 2 g

Figure 2 shows the roles of *R* and *d*_*f*_ in ln (N) and *A* levels at constant *W*_*f*_ = 2 g. According to Fig. 2a, low *N* is observed by the high values of *R* and *d*_*f*_, but *N* increases when *R* and *d*_*f*_ decrease. So, the density and size of nanoparticles inversely affect the number of particles in polymer nanocomposites at a constant filler concentration. The small nanoparticles with low density produce a large number of nanoparticles in nanocomposites, while the big and dense nanoparticles make few particles. Accordingly, the aggregates/agglomerates significantly decrease the number of nanoparticles in nanocomposites at a constant filler concentration.

Figure 2b illustrates the effects of *R* and *d*_*f*_ parameters on the total surface area of nanoparticles (*A* in *m*^2^) at *W*_*f*_ = 2 g. The surface area of nanoparticles is assumed as the interfacial area between polymer and nanoparticles transferring the stress from matrix to nanoparticles. The stress may be efficiently transported from polymer to nanoparticles to improve the mechanical properties, when the interfacial area is big enough [39, 40]. As observed in Fig. 2b, the largest interfacial area is achieved by the smallest ranges of *R* and *d*_*f*_. It is also interesting that only 2 g of isolated and well-dispersed nanoparticles with *R* = 10 nm and *d*_*f*_ = 2 g/cm^3^ produce about 250 m^2^ interfacial area with polymer matrix. However, the interfacial area reduces by increasing the size and density of nanoparticles and *A* below 50 m^2^ is obtained at *R* > 40 nm and *d*_*f*_ > 3 g/cm^3^. The significant difference between the interfacial areas at different particle sizes indicates that the nanoparticle size is an important parameter in nanocomposites. The large nanoparticles cause small interfacial area which deteriorates the significant advantage of nanoparticles in nanocomposites. It should be noted that the filler concentration in nanocomposites may not be too high compared to micro-composites, but the extraordinary surface area of nanoparticles commonly results in the interaction between particles and aggregation/agglomeration. As a result, although the high contents of nanoparticles in nanocomposites strengthen the accumulation, the aggregation/agglomeration of nanoparticles generally occur in polymer nanocomposites at different filler concentrations which decrease the interfacial area and weaken the performance.

**Fig. 3:**
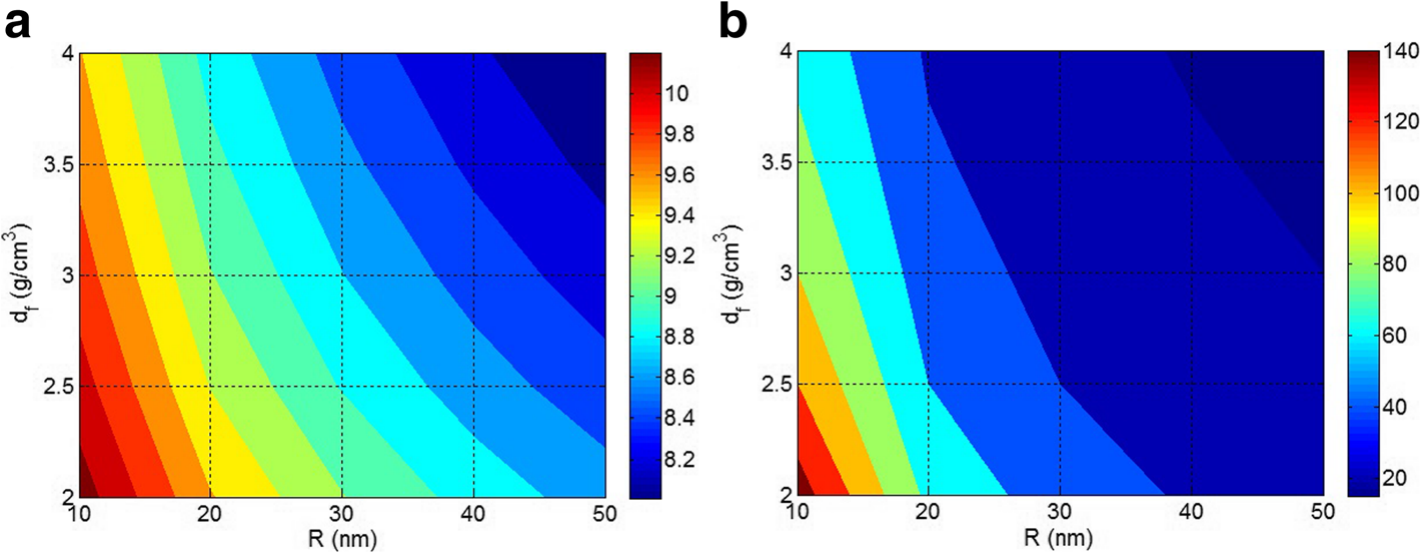
The effects of *R* and *d*_*f*_ on **a** ln (SE) with ln (m^2^ GPa) unit and **b** *A*_*c*_ (m^2^/g) at *W*_*f*_ = 2 g and *E*_*f*_ = 100 GPa

Figure 3a displays the contour plots of ln (SE) as a function of *R* and *d*_*f*_ at *W*_*f*_ = 2 g and *E*_*f*_ = 100 GPa. The stiffening efficiency of nanoparticles increases when small nanoparticles with low density are incorporated in polymer matrix, demonstrating that the nanoparticle size causes an effective role in the stiffening of nanoparticles in polymer nanocomposites. On the other hand, the aggregated/agglomerated nanoparticles deteriorate the performances of polymer nanocomposites by reduction of nanoparticle efficiency. The small nanoparticles with low density meaningfully increase the stiffness of nanocomposites through the great level of stress transferring between polymer chains and nanoparticles. A previous study in this area has explained the physics of the influence of filler radius on the stress transfer from polymer matrix to fiber using the molecular dynamics simulations [41]. However, the large and dense particles cannot introduce the high stiffness of nanoparticles to polymer matrix suggesting a composite with poor stiffness. Therefore, the characteristics of nanoparticles significantly control the properties of nanocomposites.

Figure 3b also shows the levels of *A*_*c*_ parameter at different *R* and *d*_*f*_ values at *W*_*f*_ = 2 g and *E*_*f*_ = 100 GPa. It is observed that the best *A*_*c*_ is obtained by small and low-density nanoparticles, while the worst one is produced by large and dense particles. The *A*_*c*_ value of about 140 m^2^/g is achieved by *R* = 10 nm and *d*_*f*_ = 2 g/cm^3^, while *A*_*c*_ level of less than 20 m^2^/g is shown by large particle size and high density. As a result, *R* and *d*_*f*_ parameters show negative effects on *A*_*c*_ in polymer nanocomposites. It is concluded that *A*_*c*_ parameter expressing the interfacial area of 1 g isolated nanoparticles gives the best levels by small nanoparticles. As a result, the large nanoparticles or aggregates/agglomerates cannot produce a considerable *A*_*c*_ which decreases the efficiency of nanoparticles in polymer nanocomposites. It is known that the performances of nanocomposites such as mechanical, flame retardation, and barrier properties directly relate to the interfacial area between polymer and nanoparticles [10, 42]. A large *A*_*c*_ can produce acceptable levels for nanocomposite properties by little amount of nanoparticles, due to the high interfacial area between polymer matrix and nanoparticles. Accordingly, controlling the size and density of nanoparticles are challenging in nanocomposites to create the best properties.

**Fig. 4:**
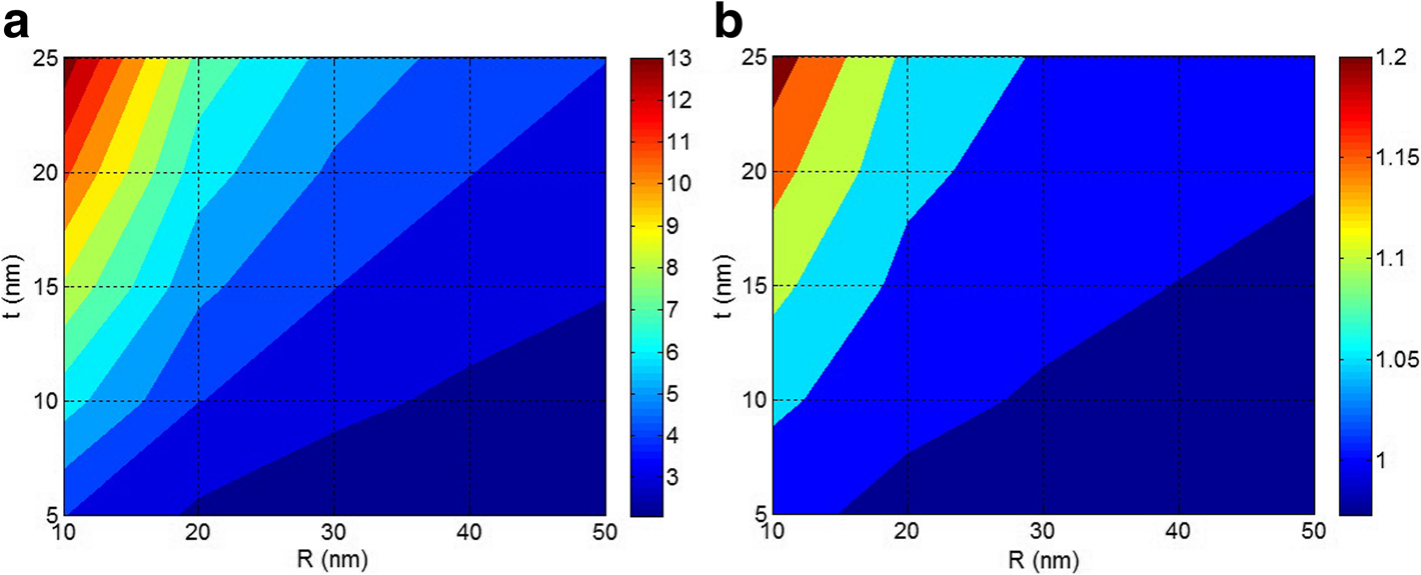
**a** *B* interfacial parameter and **b** relative tensile strength by Pukanszky model at different ranges of *R* and *d*_*f*_ and constant *σ*_*i*_/*σ*_*m*_ = 5 and *ϕ*_*f*_ = 0.02

Now, the effects of nanoparticle and interphase sizes on the interfacial/interphase properties and tensile strength of nanocomposites are explained by the proposed equations. Figure 4 illustrates the effects of *R* and *t* on *B* interfacial parameter and tensile strength by Pukanszky model (Eq. 6) at *σ*_*i*_/*σ*_*m*_ = 5 and *φ*_*f*_ = 0.02. Based on Fig. 4a, the *B* level of 13 is obtained by the smallest nanoparticles and the thickest interphase. Also, *B* decreases to below 3 when the size of nanoparticles grows to about 40 nm and the interphase thickness decreases to less than 10 nm. Therefore, the sizes of nanoparticles and interphase play dissimilar roles in *B* parameter. Also, it should be noted that the small nanoparticles without formation of a strong interphase cannot give a high *B* in polymer nanocomposites. On the other hand, a thick interphase (*t* = 25 nm) surrounding the big nanoparticles (*R* = 50 nm) only improves the *B* parameter to about 4. As a result, both nanoparticle and interphase dimensions are important to obtain a high level of *B* in nanocomposites. However, at a constant level of interphase thickness, the growth of nanoparticle size by aggregation/agglomeration decreases *B* parameter demonstrating the negative effects of aggregates/agglomerates on the interfacial/interphase properties.

Figure 4b also shows the effects of *R* and *t* parameters on the tensile strength of nanocomposites by Pukanszky model. It is observed that small nanoparticles and thick interphase improve the strength of nanocomposites. However, a poor strength is observed by big particles and thin interphase. Therefore, both *R* and *t* parameters affect the tensile strength of nanocomposites. Moreover, it is found that the strength of nanocomposites reduces when the size of nanoparticles grows, due to aggregation/agglomeration. Accordingly, it is essential to isolate and disperse the nanoparticles in polymer matrix at small size to achieve the best performances. Since nanoparticles naturally tend to aggregation/agglomeration, modification of their surface or functionalization of polymer chains can prevent the accumulation [19, 43, 44].

**Fig. 5:**
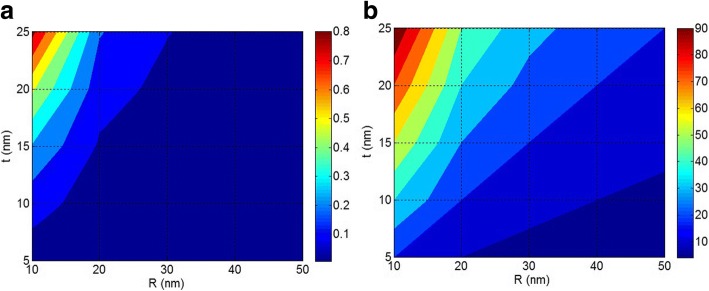
The correlation of **a** *ϕ*_*i*_ and **b** *a* interphase parameter to *R* and *t* parameters at *ϕ*_*f*_ = 0.02, *E*_*f*_ = 100 GPa, and *E*_*i*_ = 50 GPa

Figure 5 depicts the dependences of interphase volume fraction (*φ*_*i*_) and *a* interphase parameter on *R* and *t* parameters at *φ*_*f*_ = 0.02, *E*_*f*_ = 100 GPa, and *E*_*i*_ = 50 GPa. According to Fig. 5a, the smallest nanoparticles and the thickest interphase give the highest level of *φ*_*i*_ as 0.8 which significantly reinforces the nanocomposite. This level of *φ*_*i*_ is more than *φ*_*f*_ demonstrating the effective roles of *R* and *t* parameters in the performances of nanocomposites. Furthermore, *φ*_*i*_ decreases to about 0 at *R* > 30 nm, i.e., a thick interphase (*t* = 25 nm) cannot make a high *φ*_*i*_ in polymer nanocomposites when large nanoparticles are incorporated in the polymer matrix. This occurrence shows the significant role of nanoparticle size in the formation of interphase regions. So, the size of nanoparticles considerably changes the interphase properties revealing that the aggregation/agglomeration of nanoparticles mostly decreases the interphase concentration which causes poor modulus and strength in nanocomposites [5, 45]. It should be mentioned that the interphase regions may overlap in the systems containing high filler concentration. Therefore, the expressed equation for *φ*_*i*_ (Eq. 10) is reasonable for normal nanocomposites containing low filler content.

Figure 5b also shows the effects of *R* and *t* levels on *a* interphase parameter. *a* increases by small nanoparticles and thick interphase, whereas it gives less values (less than 10) at *R* > 40 nm and *t* < 10 nm. This evidence reveals that *a* depends on both *R* and *t* parameters. Since a high *a* parameter improves the Young’s modulus of nanocomposites [38], small nanoparticles and thick interphase are desirable for nanocomposites performances. According to Fig. 5b, the aggregates/agglomerates of nanoparticles (high *R*) produce slight *a* event by thick interphase. This occurrence indicates that a strong interphase cannot give a great *a* or high modulus when the nanoparticles are aggregated/agglomerated in nanocomposites. As a result, the aggregates/agglomerates of nanoparticles cause negative effects on the properties of polymer nanocomposites. Based on the mentioned remarks, the aggregation/agglomeration weakens the benefits of nanoparticles and properties of interface/interphase; therefore, the nanoparticles cannot present a strong reinforcement in polymer nanocomposites.

## Conclusions

The effects of filler size and density as well as interphase thickness on the characteristics of nanoparticles and the interface/interphase properties were studied by simple equations. Also, the aggregates/agglomerates of nanoparticles were assumed as large particles and their influences on the interphase parameters and the tensile strength of nanocomposites were discussed. The small size and low density cause significant levels for number, surface area, stiffening efficiency, and specific surface area of nanoparticles. Only 2 g of small and well-dispersed nanoparticles (*R* = 10 nm) with *d*_*f*_ = 2 g/cm^3^ can produce about 250 m^2^ interfacial area with polymer matrix. On the other hand, big size and aggregates/agglomerates weaken the positive attributes of nanoparticles in nanocomposites. Small nanoparticles and thick interphase present the high levels for *B* parameter, tensile strength, interphase volume fraction, and *a* interphase parameter. *B* decreases to below 3 when the size of nanoparticles grows to about 40 nm and the interphase thickness reduces to less than 10 nm. However, *B* = 13 is obtained by the smallest nanoparticles (*R* = 10 nm) and the thickest interphase (*t* = 25 nm). This occurrence confirms that the interfacial/interphase properties depend on the nanoparticle size beside the interfacial interaction/adhesion. Additionally, large nanoparticles produce low interfacial/interphase properties and poor tensile strength even at high interphase thickness revealing the main role of particles size. The smallest nanoparticles and the thickest interphase give the highest level of *φ*_*i*_, while *φ*_*i*_ decreases to about 0 at *R* > 30 nm. This evidence demonstrates that only a thick interphase (*t* = 25 nm) cannot make a high *φ*_*i*_ when large nanoparticles or aggregates/agglomerates are present in nanocomposites. Accordingly, the aggregated/agglomerated nanoparticles negatively affect the interfacial/interphase properties and tensile strength of polymer nanocomposites.
